# Supplementary material for: Lack of Electron Acceptors Contributes to Redox Stress and Growth Arrest in Asparagine-Starved Sarcoma Cells
Source: Cancers (Basel). 2021 Jan 22;13(3):412. doi: 10.3390/cancers13030412 (PMC7865502; doi:10.3390/cancers13030412)
Supplement: Supplementary file 1 [file cancers-13-00412-s001.zip › cancers-1072355-supplementary materials-xml/Spplementary Figures.pdf]

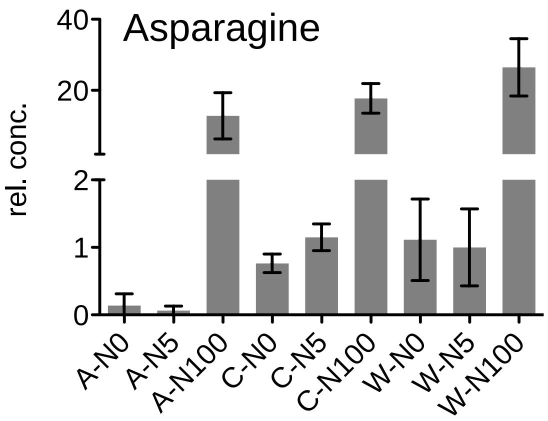

Figure S1. Relative asparagine content in shASNS (A), shLuc (C) and wild-type (W) cells grown in medium with 0 mg/L asparagine (N0), 5 mg/L asparagine (N5) and 100 mg/L asparagine (N100). Supplementation with 5ng/mL asparagine did not reverse the effects of the shASNS knockdown. Supplementation with 100ng/ml asparagine raised asparagine content in shASNS, shLuc and wild-type cells to supraphysiological levels.

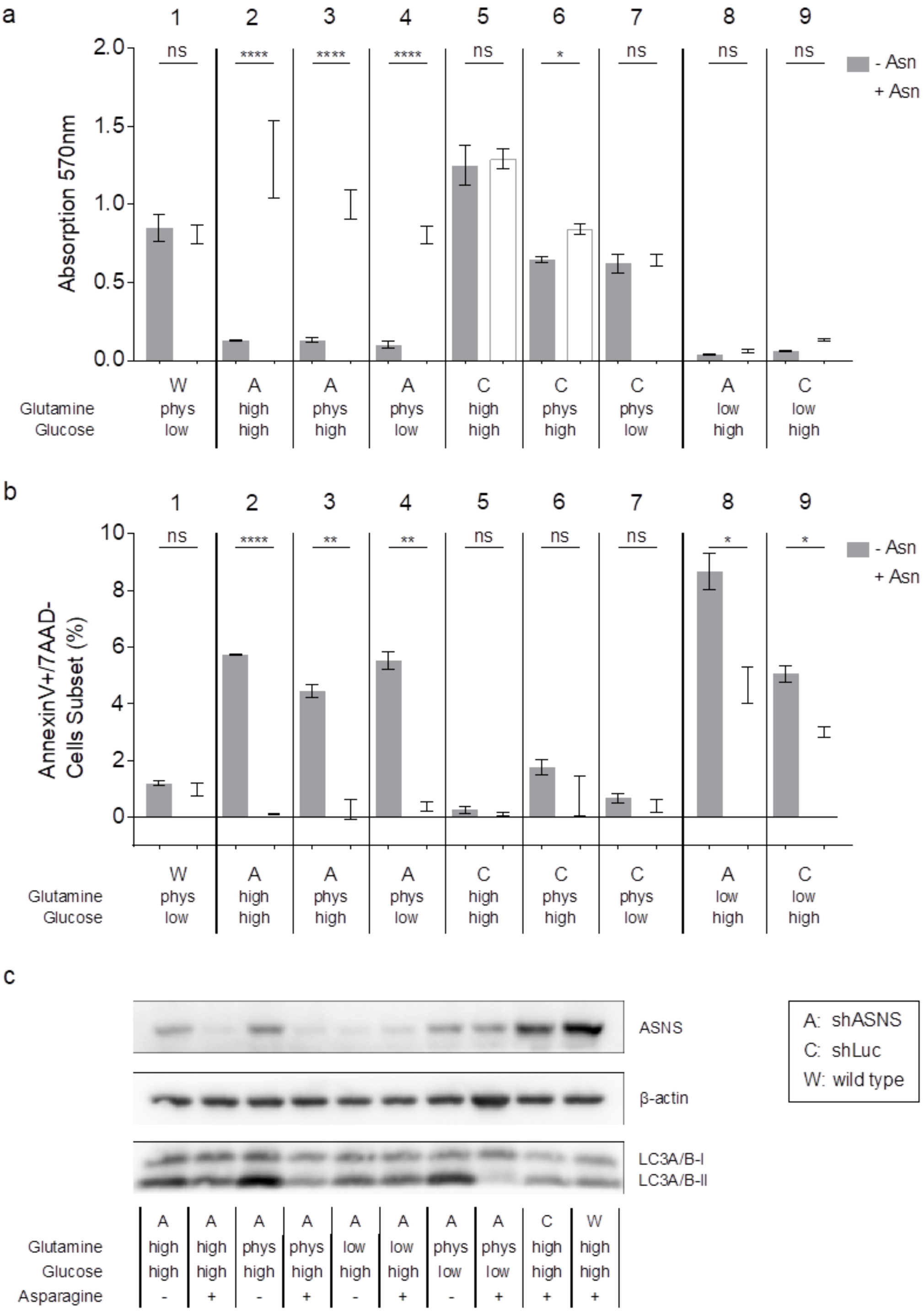

Figure S2. Impact of asparagine starvation on proliferation, apoptosis and autophagy of mouse sarcoma cells. The effects of asparagine deprivation were investigated in shASNS mouse sarcoma cells (A), shLuc mouse sarcoma cells (C) and wild-type mouse sarcoma cells (W) in the context of high, physiological and low glutamine (584.6 mg/L, 73.1 mg/L and 7.3 mg/L, respectively) as well as high and low glucose (4.5 g/L and 0.5 g/L, respectively) concentrations. Cellular asparagine deprivation by shASNS knockdown and culture in asparagine-free medium (**a**, 2–4) reduced proliferation and (**b**, 2–4) increased apoptosis in the context of low/high glucose and physiological/high glutamine concentrations. In medium with low glutamine content, both shASNS cells (A) and shLuc control (C) cells exhibited (**a**, 8–9) low proliferative activity and contained (**c**, 8–9) higher proportions of Annexin V^+/7AAD$^-$ apoptotic cells. Supplemental asparagine partially rescued the effects of glutamine deprivation on apoptosis, but not on proliferation. (**c**) Asparagine depletion increased LC3I/II processing in low/high glucose and physiological/high glutamine conditions. There was no effect of asparagine deprivation on LC3I/II conversion in low-glutamine conditions. (**a**–**c**) Exogenous asparagine (5 mg/L) reversed the effects of asparagine depletion on proliferation, apoptosis and autophagy in low/high glucose and physiological/high glutamine conditions. Please see figure S3 for representative FACS plots of Annexin V/7AAD-stained cells. Data were evaluated for statistical significance by unpaired T-tests (ns $p \geq 0.05$, * $p < 0.05$, ** $p < 0.01$, *** $p < 0.001$, **** $p < 0.0001$).

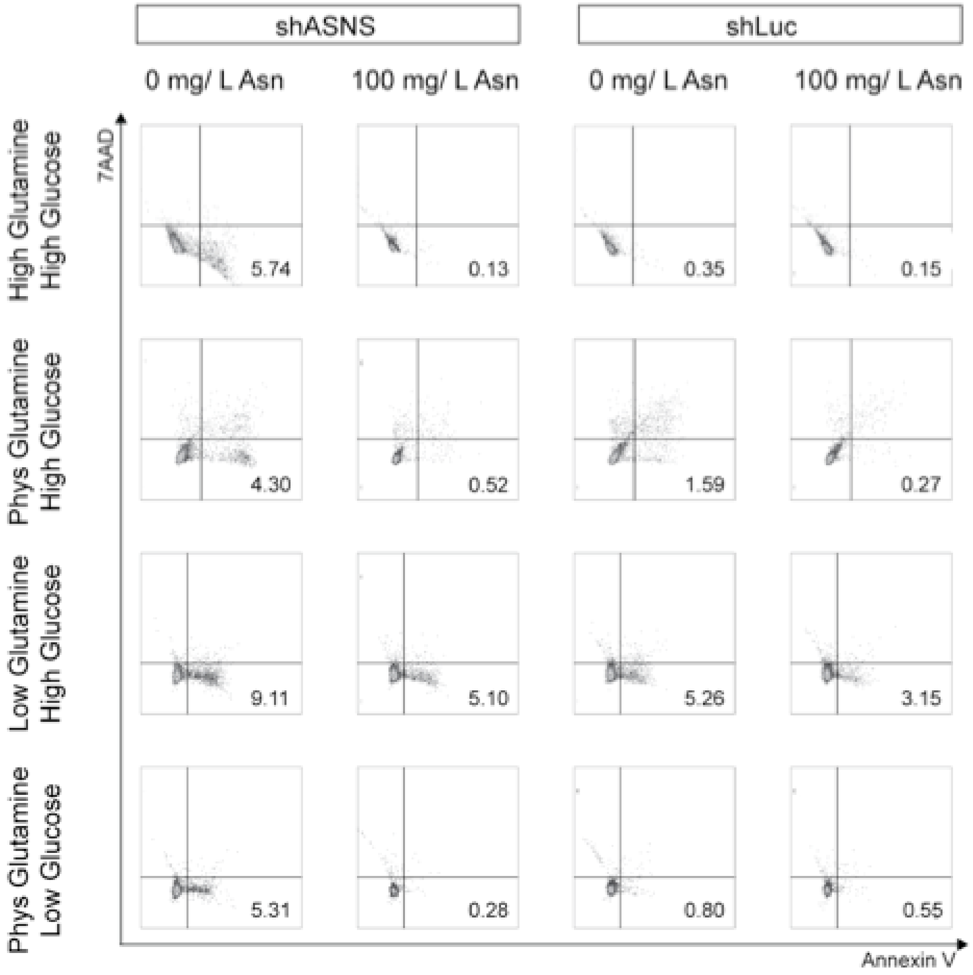

Figure S3. Higher percentage of Annexin V positive/ 7AAD negative apoptotic cells among asparagine-starved mouse sarcoma cells. Asparagine depletion was achieved by shASNS knockdown and culture in asparagine-free medium. Pro-apoptotic affects were investigated by Annexin V staining in (top row) high glucose (4.5 g/L)/ high glutamine (584.6 mg/L), (upper middle row) high glucose (4.5 g/L)/ physiological glutamine (73 mg/L), (lower middle row) high glucose (4.5 g/L)/ low glutamine and (bottom row) low glucose (0.5 g/L)/ physiological glutamine (73.1 mg/L) conditions. (top, upper middle and bottom row) Asparagine depletion raised the percentage Annexin V^+/ $7AAD^-$ apoptotic cells in low/ high glucose and physiological/ high glutamine conditions. This was reversed by exogenous addition of asparagine. (lower middle row) In medium with low glutamine content (7.3 mg/L), shASNS sarcoma cells contained many Annexin V^+/ $7AAD^-$ apoptotic cells. Supplemental asparagine partially rescued the effects of glutamine deprivation on apoptosis.

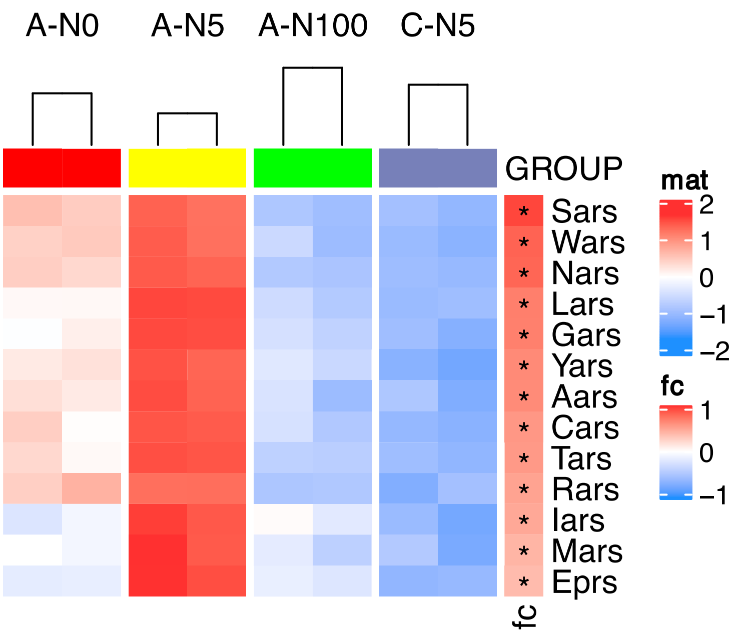

Figure S4. Differential expression of transcripts involved in aminoacyl tRNA biosynthesis in shASNS cells grown in medium containing 0 mg/L or 5 mg/L asparagine compared to shASNS cells grown in medium with 100mg/L asparagine and shLuc control cells grown in medium with 5 mg/L asparagine. Aminoacyl tRNA biosynthesis was one of the three top pathways enriched in asparagine-depleted cells as determined by pathway analyses using the KEGG database.

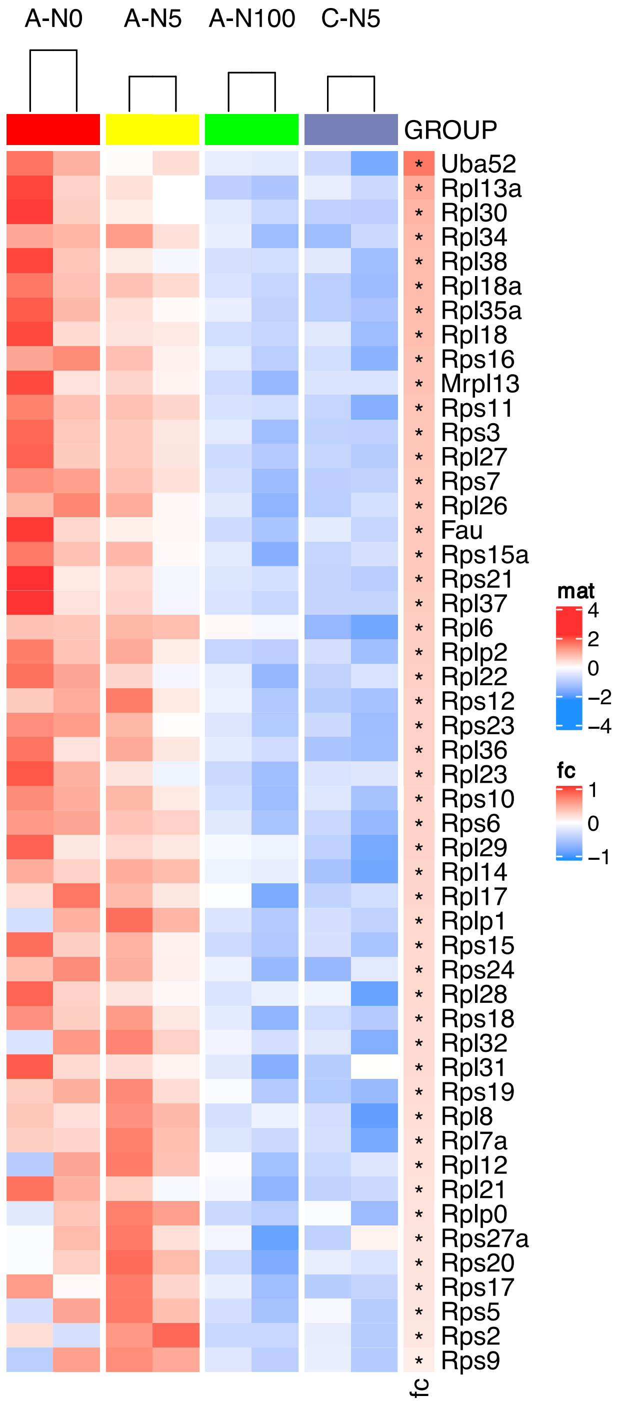

Figure S5. Differential expression of transcripts involved in ribosomes in shASNS cells grown in medium containing 0 mg/L or 5 mg/L asparagine compared to shASNS cells grown in medium with 100mg/L asparagine and shLuc control cells grown in medium with 5 mg/L asparagine. Ribosome was one of the three top pathways enriched in asparagine-depleted cells as determined by pathway analyses using the KEGG database.

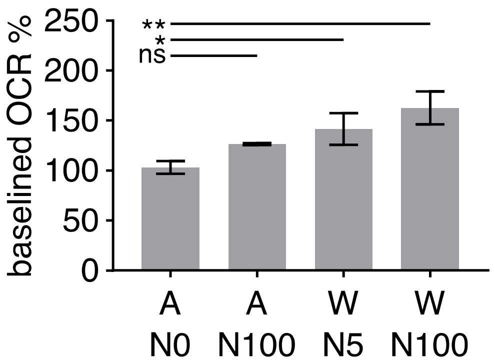

Figure S6. Lower oxygen consumption rates in asparagine-depleted cells. Oxygen consumption rates (OCR) were determined by seahorse measurements. OCR was lower in shASNS mouse sarcoma cells grown in asparagine-free medium (A-0) compared to wild-type cells grown in medium with physiological or excess asparagine content (W-N5, W-N100). Data were evaluated for statistical significance by one-way ANOVA with Tukey's post-hoc test.

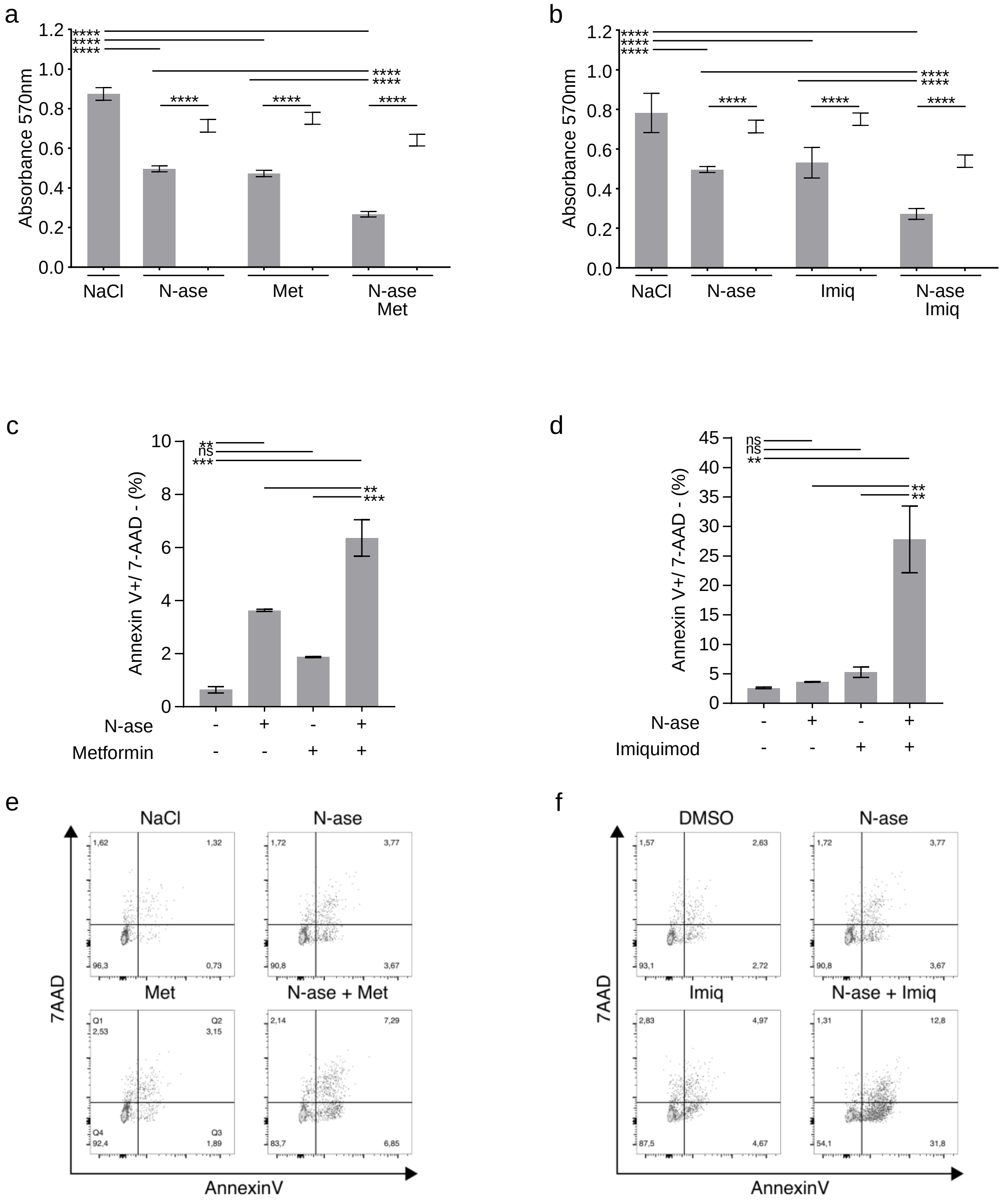

Figure S7. Synergistic growth-inhibitory and pro-apoptotic effects of complex 1 inhibitors and asparaginase on human RD rhabdomyosarcoma cells. Complex 1 inhibitors (**a**,**c**,**e**) metformin and (**b**,**d**,**f**) imiquimod augmented the (**a**–**b**) growth inhbitory and (**c**–**f**) pro-apoptotic effects of asparaginase on RD cells. (**a**–**b**) The anti-proliferative effects of metformin (1 mM), imiquimod (20 µM) and asparaginase (0.3 U/ml), alone and in combination, were reversed by addition of pyruvate. Combinatorial treatment with (**c**,**e**) metformin (2 mM) and (**d**,**f**) asparaginase (1 U/ml) or imiquimod (50 µM) and asparaginase (1 U/ml) deepened the pro-apoptotic effects of each chemical alone. (**e**,**f**) Data were evaluated for statistical significance by one-way ANOVA with Tukey's post-hoc test (ns $p \geq 0.05$, * $p < 0.05$, ** $p < 0.01$, *** $p < 0.001$, **** $p < 0.0001$).

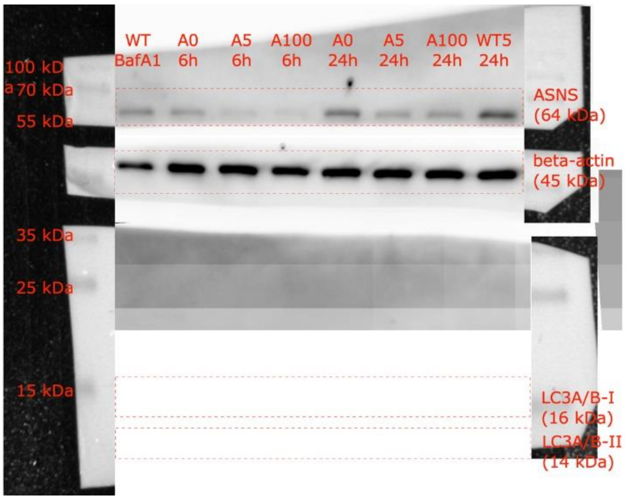

	ASNS	beta-actin
A0 6h	9085,702	26835,907
A5 6h	4343,719	27695,492
A100 6h	2164,991	24111,593
A0 24h	17678,312	27829,543
A5 24h	9946,087	28393,099
A100 24h	9883,602	26292,886
WT5 24h	23091,132	28363,078

Figure S8. Raw data of Figure 2**d**.

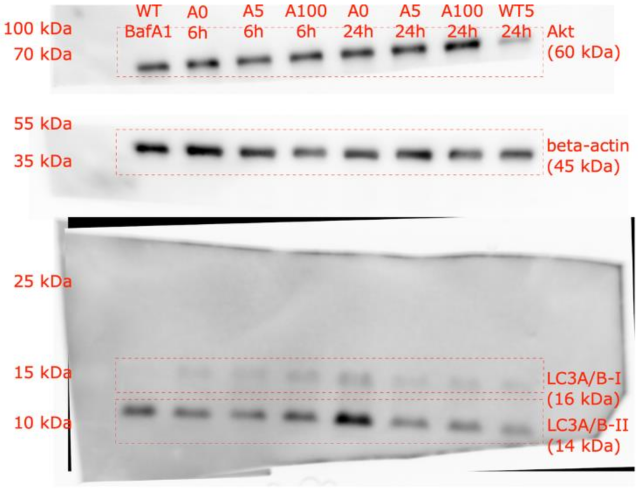

	beta-actin	LC3A/B II
A0 6h	22790,936	8351,258
A5 6h	17546,744	6770,409
A100 6h	11648,773	10358,38
A0 24h	14086,016	22191,421
A5 24h	17643,794	7530,238
A100 24h	12266,187	7158,652
WT5 24h	12603,066	3189,175

Figure S9. Raw data of Figure 2**e**.

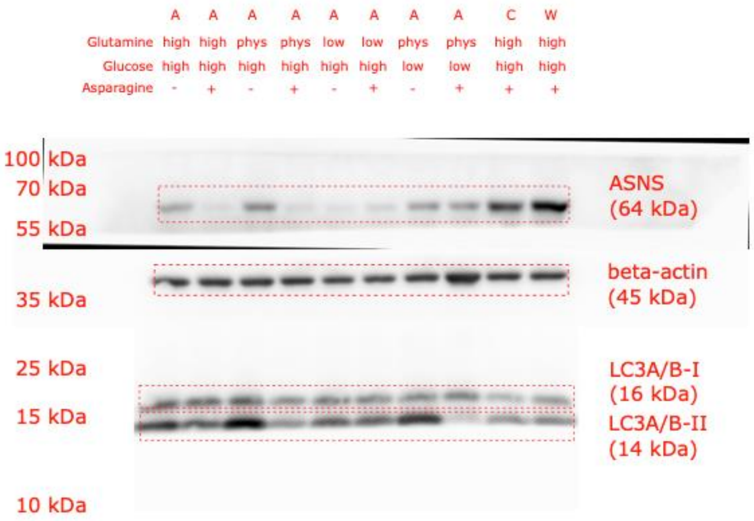

Glutamine	Glucose	Asparagine	ASNS	beta-actin	LC3A/B II
high	high	-	5170,995	15839,501	13451,279
high	high	+	711,335	18308,693	11371,622
phys	high	-	6795,309	18864,865	20094,007
phys	high	+	701,749	17432,087	7735,945
low	high	-	733,335	14360,43	10289,48
low	high	+	1290,477	13839,309	9997,551
phys	low	-	5183,581	16654,794	15000,572
phys	low	+	5231,874	24577,35	2811,418
high	high	+	16278,785	16857,966	5965,853
high	high	+	22349,049	16480,016	6884,095

Figure S10. Raw data of Figure S2.
